# More ethics in the laboratory, please! Scientists’ perspectives on ethics in the preclinical phase

**DOI:** 10.1080/08989621.2023.2294996

**Published:** 2024-01-18

**Authors:** Paola Buedo, Eugenia Prieto, Jolanta Perek-Białas, Idalina Odziemczyk-Stawarz, Marcin Waligora

**Affiliations:** a Research Ethics in Medicine Study Group (REMEDY), Jagiellonian University Medical College, Krakow, Poland; b Instituto de Diversidad y Evolución Austral (IDEAus), Consejo Nacional de Investigaciones Científicas y Técnicas (CONICET), Puerto Madryn, Argentina; c Institute of Sociology and Center of Evaluation and Public Policy Analysis, Jagiellonian University, Poland and Warsaw School of Economics, Warsaw, Poland; d Doctoral School in the Social Sciences (Sociological Sciences), Jagiellonian University, Krakow, Poland

**Keywords:** Preclinical research, bioethics, integrity, biotechnology, biomedical research

## Abstract

In recent years there have been calls to improve ethics in preclinical research. Promoting ethics in preclinical research should consider the perspectives of scientists. Our study aims to explore researchers’ perspectives on ethics in the preclinical phase. Using interviews and focus groups, we collected views on ethical issues in preclinical research from experienced (*n* = 11) and early-stage researchers (ESRs) (*n* = 14) working in a gene therapy and regenerative medicine consortium. A recurring theme among ESRs was the impact of health-related preclinical research on climate change. They highlighted the importance of strengthening ethics in relations within the scientific community. Experienced researchers were focused on technicalities of methods used in preclinical research. They stressed the need for more safeguards to protect the sensitive personal data they work with. Both groups drew attention to the importance of the social context of research and its social impact. They agreed that it is important to be socially responsible – to be aware of and be sensitive to the needs and views of society. This study helps to identify key ethical challenges and, when combined with more data, can ultimately lead to informed and evidence-based improvements to existing regulations.

## Introduction

In recent years, there have been calls to improve ethics in preclinical research (Dodson and Pawlik 2014; Landis et al. 2012; Yarborough et al. 2018). Poor translation to the clinical research phase and the replicability crisis are some of the notorious issues motivating these calls (Haslberger et al. 2023; Karp and Reavey 2019; Kimmelman and Henderson 2016; Yarborough et al. 2018). The use of same-sex animals for certain types of research has been shown to be problematic for translating research to diverse populations (Karp and Reavey 2019; Shah, McCormack, and Bradbury 2014). Other examples include lack of blinding of treatment allocation to animals, exclusion of animals because of unexpected results, and mischaracterization of the utility of a drug (i.e., a drug for a chronic human disease is tested on animals during an acute illness) (Kimmelman and Henderson 2016; Macleod et al. 2015; Wang et al. 2022). However, discussions and training on research ethics are not frequent in the preclinical research environment (Hildt et al. 2022; Laas et al. 2022). This could lead to ethical challenges in preclinical research being overlooked, but also to a lack of awareness to identify other challenges that may be subtle and difficult to recognize (Dranseika, Piasecki, and Waligora 2016; Jongsma and Bredenoord 2020).

In addition, some preclinical developments in health-related biotechnology could have an impact on society and raise new ethical concerns. They could change the way society perceives and understands health and disease, increase discrimination or redefine human identity (Buedo et al. 2023a; Jongsma and Bredenoord 2020; Torres-Padilla et al. 2020; van Delden and Bredenoord 2015). For instance gene therapy could have an impact on the identity of certain groups, such as deaf people, many of whom do not see themselves as having a disability, but rather see deafness as a personal characteristic that is part of their identity. If somatic gene therapy could play a role in “treating” these diverse functions through genes, then diverse functions could be seen simply as a genetic problem and could impact on the identity of those who do not see themselves as having an abnormality (Buedo et al. 2023a).

Promoting ethics in preclinical research should take into account the perspectives of scientists since scientists have to deal with these issues on a daily basis (Yarborough et al. 2018). Exploring how scientists perceive the relevance of ethics to their work and their responsibilities as members of society is crucial for efforts to promote ethical behavior in preclinical research, and moreover, to foster discussion in this research phase (Linville et al. 2023; Wäscher, Biller-Andorno, and Deplazes-Zemp 2020).

Using qualitative methods, we collected views on ethical issues in preclinical, laboratory research from experienced and early-stage researchers in a consortium working on developing gene therapy. We focused on this group of researchers because they work in the preclinical phase of research, and also because they are involved in genetic research, which adds a layer of complexity to the observation and analysis of ethical challenges in this phase of research.

Our aim is to explore the perspective of researchers at different stages of academic careers and gain insight into their approach to ethics in biotechnologies in the early stage of development.

## Methods

**Figure 1.**
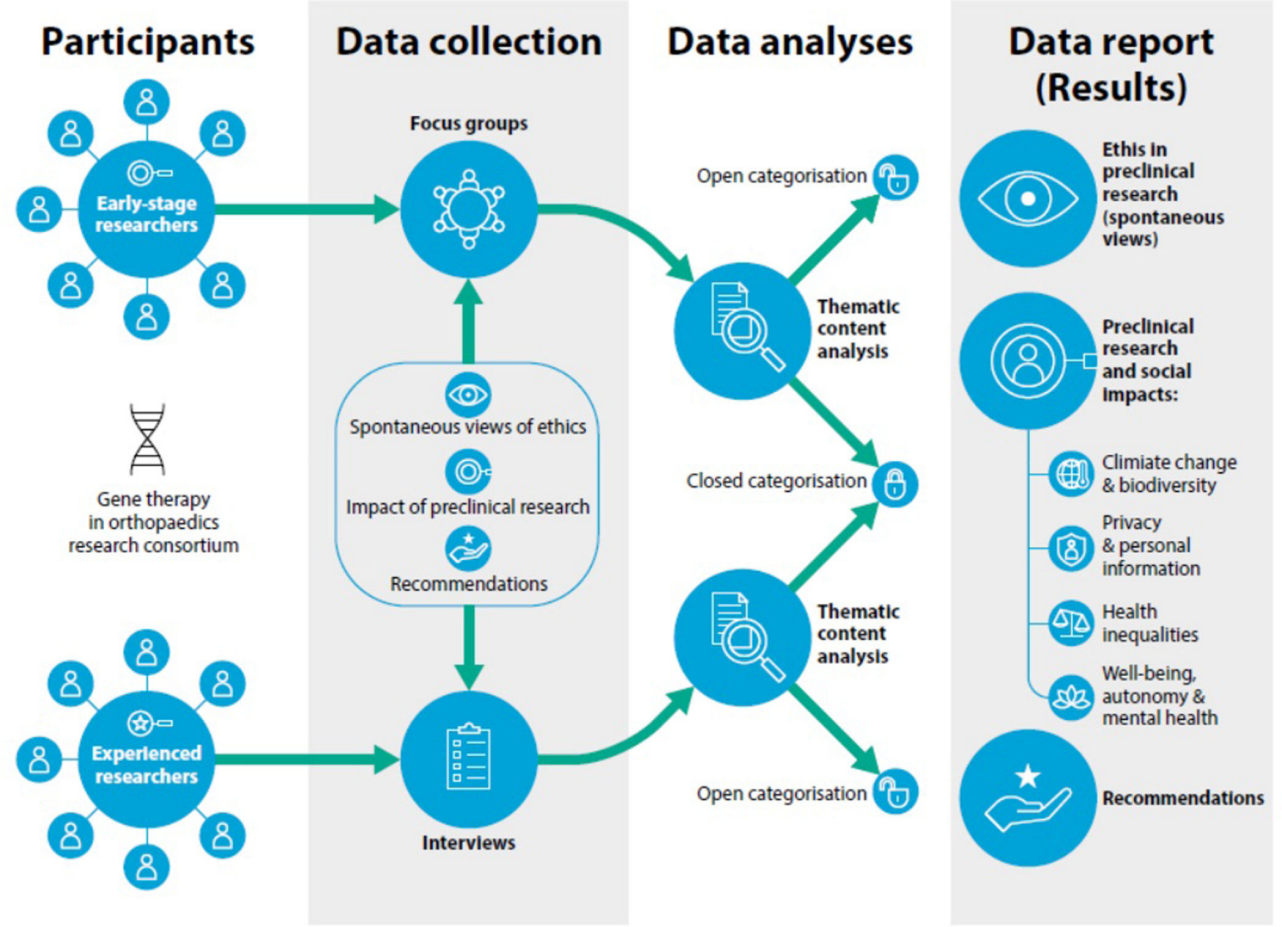
An illustrative synthesis of the methods used in this study.

To fulfil the aims of the study we applied a qualitative research strategy (Figure 1). We chose two different qualitative techniques, focus groups and individual interviews, to better adjust to the research participants’ profiles. Considering their characteristics, career situations and ways of acquiring and transmitting knowledge and information, we divided participants into two research groups. The first research group were early-stage researchers (ESRs) who had just started their career and the second research group were much more experienced experts in the field. However, in both, we share the same goal and aim to cover the same topics/areas of research interest.

We use the comprehensive consolidated criteria for reporting qualitative research (COREQ) to report our research (Tong, Sainsbury, and Craig 2007) (checklist available in Supplementary Material S1).

### Participants

All participants (*n* = 25) were recruited from a consortium created with a Horizon 2020 Marie Skłodowska-Curie grant (agreement No. 955335). The consortium focused on the preclinical development of gene therapy in orthopedic regenerative medicine. Project’s research topics include cell delivery and gene modulation efficiency, tissue/organ delivery tools, repair in tissue and organ culture, and in vivo imaging of regeneration and gene therapy efficacy.

The first group participated in focus group meetings and consisted of fourteen ESRs from Brazil (2), India (2), Iran (2), Italy, Spain, Taiwan, Germany, China, the Netherlands, Chile and Egypt. Ten were women and four were men. They currently work in the Netherlands (4), Switzerland (2), Sweden (2), Denmark (2), Finland, Romania, Germany and Portugal, in universities (10) and companies (4).

The second group participated in individual interviews and were eleven experienced researchers working as Principal Investigators in the Netherlands (3), Switzerland (2), Sweden, Denmark, Finland, Romania, Germany and Portugal, in universities (7) and private companies (3). There were seven men and four women.

### Data collection

We collected data using two different techniques: focus group discussions and semi-structured individual interviews between October 2021 and September 2022.

#### Focus groups

The focus groups consisted of five consecutive meetings between October 2021 and May 2022. The topics discussed were research ethics and integrity in the preclinical research that they were conducting, the impact of the research and their recommendations for improving ethics and integrity at this phase. The choice of focus group as a research method for ESRs group results from the desire to examine how a comprehensive concept such as ethics develops in discussions between people whose attitudes have not yet been strongly established by the influence of the research environment. We also wanted to capture the initial differences in the level of familiarization with this topic and develop the knowledge about it during subsequent meetings. The complementary aim of focus group meetings held with ESRs was to work together on recommendation how to embed ethics into laboratory research (Buedo et al. 2023b). ESRs share other educational activities as a group, thus such workshops were matched with their curriculum.

Focus group discussions were conducted by PB (one ESR from the consortium, female, MD, MA). Each meeting lasted a maximum of 90 minutes. As the ESRs were located in different countries, the FGs were conducted online. A guide for each FG was designed (Supplementary Material S2) and discussed among the research team conducting this study. One focus group was piloted with ten ESRs working in the study area but not being the part of the consortium. Technical support was provided by an ESR from outside the consortium (IOS), who was present at each FG.

#### Interviews

Semi-structured interviews performed with experienced researchers who work in different institutional contexts were treated as expert interviews. The aim was to have an in-depth conversation regarding the interviewee’s knowledge and opinion of the state of ethics and integrity in the preclinical phase. The guide consists of open-ended questions related to research ethics, integrity and bioethical challenges in the preclinical phase, as well as the impact of the research and its recommendations for improving ethics and integrity in this phase. The semi-structured design ensured consistency in the topics discussed by all participants, but also allowed participants to raise or emphasize issues different from those suggested. Separate meetings with experienced researchers allowed them to share their experience and express their views more freely, without having to confront them with the positions of other members of the academic community. The individual interviews did not include an educational supplement.

Interviews were conducted between July and September 2022 and lasted between 45 and 70 minutes. They were conducted in English and took place either at a location chosen by the participant (3) or online via a video call platform (8). The interviewer (PB) and the participants had brief prior contact at two consortium meetings. The interview guide (Supplementary material S3) was developed and discussed among the research team conducting this study. The interview was piloted with two researchers working in the study area but outside the consortium.

### Data analyses

Interviews and focus groups were recorded, transcribed verbatim and pseudonymised.

Transcriptions were read several times to familiarize ourselves with the data. Transcriptions were entered into MAXQDA software for analysis. We analyzed all data using thematic content analysis (Bergin 2018; Green and Thorogood 2018). The coded categorization (PB, EP) was developed according to the research objectives of the study. In doing so, we combined a closed and open approach to codes, meaning that we defined only some of the codes prior to analysis (Taylor, Bogdan, and DeVault 2015). The closed categorization related to research impact on autonomy, privacy and personal information, climate change, health inequalities, social well-being and mental health. Open codes were based on the data from the transcriptions of spontaneous views on ethics in preclinical research and recommendations. As the interview and focus group data were analyzed separately, once the coding was complete, we established a relationship between the categories in order to further present and discuss our findings.

### Ethical considerations

The protocol, informed consent form, the General Data Protection Regulation (GDPR) form and participant information page were approved by the Bioethics Committee of the Jagiellonian University, Krakow, Poland (No. 1072.6120.209.2021–29/09/2021). Participants were informed individually by e-mail about the aims of the study, what their participation would involve, why they were invited, the risks and benefits of their participation, and that the sessions would be recorded. We also emailed them the GDPR form and the informed consent form. We explained that the information obtained from the interviews and focus groups would only be used for research purposes and, if published, all data would be anonymized (Daniels et al. 2019; Sim and Waterfield 2019), so there would be no way to link opinions to a specific person.

## Results

We report the findings in three sections according to themes and categories that we developed during the analysis phase of the research (Table 1). Section one summarizes participants’ spontaneous views on what is ethically important in preclinical research. Section two presents researchers’ views on the different types of impacts that preclinical research has or could have. Finally, section three provides recommendations from both groups of researchers on how to improve ethics in preclinical biotechnology research.

### Spontaneous views on ethics in preclinical research

There were two themes that both experienced and early-stage researchers spontaneously associated with ethics in preclinical research: animal experimentation and the use of human biological material and how it is obtained. Both groups also agreed that even though their work is based in a laboratory setting, it is important to be sensitive to the needs and views of society, to be socially responsible in three senses: to let people know what they are doing, to be mindful of the research funding source and to be aware that what they do has consequences, and therefore to consider the social impact of research.

Experienced researchers associated ethics with procedures and requirements of the institutions where they conduct research, with guidelines and with external approval. Some of them expressed that preclinical research needs “standard ethics,” but if the research project is granted by a highly recognized institution, few expressed that there is no need to consider additional ethical issues as they relied on the institution to ask them to address particular ethical challenges if they considered it necessary. A minority mention that ethics is not needed at preclinical stage at all. Others suggest that there is already overregulation in terms of ethics in the academic context. Safety, toxicity, adverse events and long-term effects were also presented by most experienced researchers as ethically relevant topics.

Early-stage researchers related ethical issues to data production and management, such as integrity, reproducibility and security. They stressed the importance of reporting all experimental details in a publication and of publishing so-called “negative results.” Some of them mentioned authorship as an ethically sensitive topic. Furthermore, ESRs placed ethics in the context of the relationships within the scientific community, referring to improving mentoring, respecting other researchers, being able to work more collaboratively and the need for more multidisciplinary and multicultural teams. They expressed that, at the preclinical phase, it is important to take into account the potential impact of the research on people and society, rather than just focusing solely on the individual’s scientific topic. Finally, a recurring theme among ESRs was the impact of preclinical research on climate change, with in-depth discussions on waste generation, chemical treatment and sustainable research.

### Preclinical research and social impacts: the case of gene therapy in orthopaedics

The overall aim of the research consortium where participants of this study are working is to investigate the applicability of non-viral gene therapy in osteoarthritis and disc degeneration through cartilage regeneration. The societal implications of this preclinical research may be partly topic-specific. However, we have included them because some perspectives and views are general enough to be applicable to other areas of research. They may also be useful in a wider debate about ethics and integrity in preclinical research.

#### Impact on climate change and biodiversity

Scientists from both groups reflected that preclinical research produces an environmental footprint. All ESRs emphasized the footprint consequences of their research activities, with the issue being raised repeatedly. On the other hand, five experienced researchers were not convinced that preclinical research has an impact on climate change, or that there are other major players responsible for the “real” environmental impact, such as big pharmaceutical companies. ESRs and experience researchers who thought there was an impact cited the use of plastics in preclinical research, the production of chemical and biological waste, the energy used to keep the temperature of some biological samples constant, and the large amount of water used in experiments. ESRs also mentioned that scaling up a new treatment may require more infrastructure, which could generate even more footprint.

Some experienced researchers suggest that the environmental impact of preclinical research is underestimated and should be addressed, and that regulation could help make the process more sustainable. One experienced researcher mentioned the “green lab” strategy as a possible way to address this issue. In addition, some researchers in both groups felt that air travel by researchers should be reduced.

#### Impact on privacy and personal information

Some experienced researchers emphasized that personalized medicine techniques may pose some risks of donor identification. They also suggested that researchers in preclinical research work with sensitive personal data and that more safeguards are needed to protect this type of data. Some of them mentioned that details of human tissue donors should not be tracked. Conversely, seven experienced researchers were convinced that preclinical research could have no impact on or influence on privacy. ESRs did not elaborate much on this issue.

#### Impact on health inequalities

After a general question on the topic, scientists from both groups came up with the economic dimension of health inequalities. They agreed that innovative therapies can be expensive and therefore only affordable by wealthy people in developed countries. They also suggested that these types of treatment may be more efficient and therefore cheaper in the long term. Researchers suggested that these innovations should be accessible and eventually included in insurance or public health plans. Both groups agreed that it is important to discuss the use of public funding for health-related research, as people are researching treatments for rare diseases when many people are dying from prevalent diseases, such as malaria.

They mention the role that the “sex of cells” (verbatim from participants, “sex of cell lines” was what they referred to (Shah, McCormack, and Bradbury 2014)) as well as the ethnic origin and age of the biological material could affect the efficacy of the therapy in diverse populations, so these should be taken into account in advance in preclinical research.

Technical dimensions during the development of the potential therapeutics (i.e., the type of storage that would be required, the technical capacity to deliver the treatment, the technical needs for follow-up) should also be considered at the preclinical stage of research in relation to health inequalities. If more complex conditions are required to use or apply a treatment, it may be difficult to make the treatment available in all economic and cultural settings around the world.

#### Impact on social well-being, autonomy and mental health

When asked about the potential impact of their research on societal wellbeing, all participants agreed that positive results from their gene therapy research could improve the quality of life, especially in aging societies, so that the results could have an overall positive impact on global health. Both groups stated that this could also increase the overall autonomy of future patients. Patients could be more autonomous because their mobility could increase and they would be less dependent. Experienced researchers stated that increased mobility provides the opportunity for sport and exercise, which can have a positive impact on other types of illness and increase overall wellbeing. Increased mobility and the possibility of pain relief could have a positive impact on social life and mental health by preventing isolation of future patients.

Both groups also mentioned the economic burden caused by chronic diseases and believed that the potential new therapy could also have a positive impact in this area, as it could help to reduce orthopedic chronic diseases.

Regarding the negative impact that preclinical research may have in the well-being dimension, the ESRs mentioned that taking tissue from dead donors may negatively affect the emotions of the donor’s family, as some people have strong feelings against compromising the wholeness of the body. Some of the experienced researchers mentioned that new treatments involving genes may create new frictions in society. If the new treatment has adverse effects, citizens may lose confidence in other similar treatments in the future.

### Recommendations or what we can do better in health-related preclinical research

**Figure 2.**
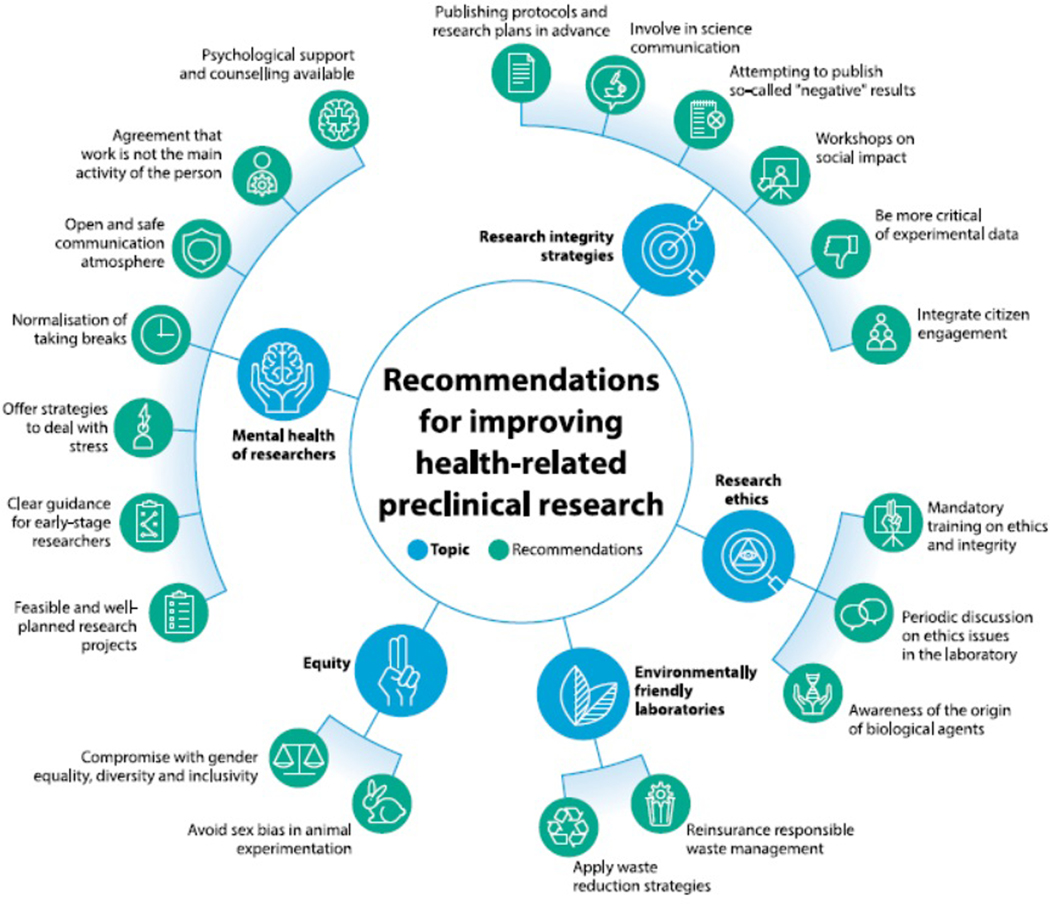
Recommendations for improving health-related preclinical research.

The majority of both groups agreed that more research integrity policies are needed, that more attention should be paid to the mental health of researchers, and that ethics training should be mandatory. ESRs were very concerned about climate change, so their recommendations were to focus on responsible laboratory waste management and waste reduction strategies. They emphasized the need to work on gender equality, diversity and inclusivity in the research process and research ecosystem. Experienced researchers mentioned that scientists working in the pre-clinical phase need to be more involved in science communication. More detailed recommendations are presented in Figure 2.

## Discussion

This article provides an overview of the perspectives and views of scientists at different stages of their careers on ethics and integrity in preclinical research.

One of the most important findings is that although most researchers participating in our study can relate to ethics and research integrity in some way, they also recognize gaps in their knowledge. Recent findings indicate a significant discrepancy between what was expected regarding ethics and what was presented in the research proposal of Horizon 2020 (Buljan, Pina, and Marušić 2021; De Waele et al. 2021; Tabarés et al. 2022). A case study conducted with scientists in the field of nanomedicine (Silva Costa et al. 2011) and an in-depth interview study with scientists in regenerative medicine research (Niemansburg et al. 2015) showed similar results. Most scientists in our study linked ethics to guidelines and legal frameworks, and they also reiterated that if an ethical issue is related to their own research, it is similar to others that already exist and have been addressed. This approach was described by Wolpe (2006), who concludes that scientists avoid thinking about ethics because they consider that their work has little to do with ethics and also that “others will make the ethical decisions” (Wolpe 2006). Jensen et al. (2011) reported data along these lines, showing that scientists perceive ethical and social issues as an external agenda that is somehow imposed on them (Jensen et al. 2011). Similarly, Wäscher, Biller-Andorno, and Deplazes-Zemp (2020) showed in an interview study that scientists emphasized that ethical issues go beyond the expertise of their professional role. They also analyzed that some interviewees expressed the idea that knowledge is morally indifferent, which was also the feeling of our respondents (Wäscher, Biller-Andorno, and Deplazes-Zemp 2020). This could be one reason why scientists in our study did not extensively address unconscious bias as has been found in another studies (Cairns et al. 2021; Davies 2019). Unconscious bias has been associated with unethical behavior, for example, research hypotheses could be framed by incorporating socio-cultural prejudices in designing experiments (Cairns et al. 2021; Davies 2019).

In contrast, Ladd et al. (2009) found that some researchers are aware that scientific processes do not take place in a vacuum and that laboratories exist in social contexts (Ladd et al. 2009). The ESRs in our study had a similar view of science and were keen to point out that although their research could be very specific and technical, they should keep in mind what they called “the bigger picture,” meaning that what they are doing has a social purpose. Moreover, ESRs were also concerned about the research impact on ecology. We found that these concerns relate to the fact that they have a clear idea that research is connected to the social and environmental contexts. This is quite different from what is usually seen in research ethics in biotechnology, and we were surprised when this topic came up. ESRs were not just concerned about these impacts, but they were informed on different strategies that could deal with this situation.

Systemic or institutional issues are mentioned by scientists as an important factor for conducting ethical research, but also for creating a friendlier working environment. Scientists participating in our study are aware that the workplace is an important factor for exercising integrity and ethics in research. Similar results were presented elsewhere (Cairns et al. 2021; Davies 2019; Solomon et al. 2022). On the other hand, ESRs in our study associate ethics and integrity with wellbeing and working in a healthy environment. During the focus groups, they often paused to analyze how their mental health affects the way they work, and how this might somehow make them less sensitive to ethical issues.

As reported in other studies, scientists are motivated to reflect on ethical issues in their work and to participate in ethical discussions and training when opportunities arise (McCormick et al. 2009; Silva Costa et al. 2011). In our study, ESRs showed interest and engagement with the ethical issues, deep reflection on integrity and their own daily experiences as scientists, and a desire to make things better. Experienced researchers were also interested and, in most cases, were available for more than an hour-long interview, stating that the questions were useful for them to reflect on issues they rarely think about. However, some of them were more reluctant to put the ethics and integrity as priority.

Our study has limitations. First, qualitative studies are prone to bias, as a different interviewer/moderator may have focus on different aspects of the participants’ interventions and the authors may have analyzed the data differently. Second, all participants and moderator/interviewer were from the same research consortium, although from different countries and with different backgrounds. Nevertheless, the sharing of a professional scenario between the facilitator/interviewer and the participants could contribute to a quicker adaptation to the situation of the interview/focus group, without much effort or calculation (Criado 1998). This is a desirable scenario to engage with the participants in order to address sensitive issues, creating a space of trust and allowing them to be more open. Third, the participants were involved in research into gene therapy for orthopedic conditions, so some of the responses here may be specific to this topic. Four, most ESRs came from the Global South, while most of experienced researchers are from the Global North. This could be another way of grouping besides career stage.

Despite its limitations, this study provides valuable information on ethics and integrity in health-related preclinical research from the perspective of scientists working in laboratories. These views help to identify key ethical challenges and, when combined with more data, ultimately lead to informed and evidence-based improvements to existing regulations.

Preclinical health-related research has an ethical dimension that impacts day-to-day work. Failure to understand the perspectives of researchers could contribute to overlooking the real needs and problems that arise in preclinical research. The more we consider this in the early stages of research, the better we can address them appropriately in the pursuit of successful science.

## Supplementary Material

More ethics Supp 1

More ethics Supp 3

More ethics Supp 2

## Figures and Tables

**Table 1.** Themes and categories developed from focus groups and interviews.

Themes	Categories in Focus Groups	Categories in interviews

1. Spontaneous views on ethics in	*Animal experimentation*	
preclinical research	*The use of human biological material and how it is obtained*	
	*Integrity*	*Institutional procedures*
	*Relationships in scientific community*	*Standard/no-need ethics*
	*Impact in society*	*Safety, toxicity and long-term effect*
	*Footprint on environment*	
2. Preclinical research and social impacts:	*Impact on privacy and personal information*	
the case of gene therapy in orthopaedics	*Impact on health inequalities*	
	*Impact on social well-being, autonomy and mental health*	
	*Impact on climate change and biodiversity*	
3. Recommendations or what we can do	*Research integrity strategies*	
better in health-related preclinical	*Ethics training*	
research	*Avoid sex bias*	
	*Equity*	*Science communication*
	*Mental health of researchers*	*Citizen engagement*
	*Environmentally friendly laboratories*	
