# Supplementary material for: More ethics in the laboratory, please! Scientists’ perspectives on ethics in the preclinical phase
Source: Account Res. Author manuscript; Available in PMC 2025 Aug 1. (PMC11778529; doi:10.1080/08989621.2023.2294996)
Supplement: More ethics Supp 3 [file NIHMS2040423-supplement-More_ethics_Supp_3.pdf]

More ethics in the laboratory, please! Scientists' perspectives on ethics in the preclinical phase

Interview Guide

---

1. Can you introduce yourself and explain how you are involved in gene therapy and regenerative medicine research?
2. Do you think that gene therapy and regenerative medicine could have ethical implications? Could you describe them? How should we deal with them?
3. Do you think that the ethical implications are different in the context of industry and academia? In what way?
4. In your opinion, what are the conditions under which gene therapy and regenerative medicine technologies could be used, or what limits should be in place?
5. Who should make decisions about the development and potential use of gene therapy and regenerative medicine technologies? For example, what should be the role of scientists, governments and citizens?
6. How do you think gene therapy and regenerative medicine research or technologies could impact, positively or negatively?

- Human autonomy	- Privacy and personal data
- Social well-being	- Health inequalities
- Mental health	- Biodiversity
- Climate change	- Ageing population

7. Could you describe what Responsible Research and Innovation (RRI) is?
8. How can RRI improve gene therapy and regenerative medicine research?
9. Do you apply all or some of the six keys of RRI in your work on gene therapy and regenerative medicine research? If so, how?
10. How could you improve the application of the six keys of RRI in your work on gene therapy and regenerative medicine research?
11. Are there any issues that have not been addressed that you would like to share/discuss?
