# Supplementary material for: More ethics in the laboratory, please! Scientists’ perspectives on ethics in the preclinical phase
Source: Account Res. Author manuscript; Available in PMC 2025 Aug 1. (PMC11778529; doi:10.1080/08989621.2023.2294996)
Supplement: More ethics Supp 2 [file NIHMS2040423-supplement-More_ethics_Supp_2.pdf]

More ethics in the laboratory, please! Scientists' perspectives on ethics in the preclinical phase

Focus groups guides

1st FOCUS GROUP GUIDE

---

1. Scene setting and ground rules (5 minutes)
1.a. Personal introduction:

My name is Paola, as you know, and I´m an Early-Stage Researcher in CARTHAGO project as all of you. Thank you so much for being here, and I'm looking forward to discussing and exchange ideas and thoughts with you. Ida will stay with us doing the technical support, so if you had any problems send her direct message and she will help you.

1.b. Outline of the research topic and purpose of the study:

Our meeting is part of one of the work packages of CARTHAGO: 2.5.

Work Packages 2.5 is about responsible research and innovation, following Horizon 2020 aims. The idea is to establish an educational framework towards the integration of ethics in research. Ethical issues of a novel biomedical intervention are identified and evaluated parallel to development of the field, rather than at the end-of-pipeline

The plan is to have several and periodical meetings to think, exchange ideas, debate different issues related to ethics.

1.c. Motivation to participate:

This meeting is for us, for all of us. As Work Package established, in these meetings we have to think about ethical issues on our research process, from our perspective. The important issue is that the perspective that matters is ours. And the framework that we start to build here will help us to be better scientists.
Also, what we start doing today is an innovative way of practice bioethics, so we are also being part of something kind of Avant Gard.

1.d. Ethics, confidentiality, and data process:

All these discussions were approved by the ethics committee of the Jagiellonian University Medical College, Cracow, Poland.

We will record this meeting. Recordings will be used only for research purposes. Data will be stored in safe place, following the EU General Data Protection Regulation (GDPR), as you already know after signing GDPR and inform consent form.

1.e. How to participate in the right way? Structure and rules

How our meetings will look like: This is an open discussion. I will ask questions, show you videos and pictures to start our conversation. You should feel free to say what you think, to present doubts that you may have, or to share some experiences and comments. We are on a safe environment here. It is important to know that there are no right or wrong answers. All points of view are important and welcomed. It is also important to agree or disagree with other participants because our goal is to hear as many as thoughts as possible.

Practical rules:
- Try to participate in every topic.
- Always talk to the group and not to each other in sub-groups.
- Raise virtual hand every time you want to say something - this will help us to organize the discussion better.

Do you have any doubts or questions?

---

2. Individual introductions (15 minutes – could be 5 minutes longer)

At the beginning, I'd like us to introduce ourselves. Some of us already did this, but we have new colleagues here so let's say few words about ourselves.

So, I will start – I am Paola, from Argentina, Italian citizen, a medical doctor, with a master in bioethics, and living in Krakow since April and working a lot in my PhD, which is about doing an ethical and real-time evaluation of non-viral gene therapy and orthopaedic regenerative medicine.

Could you please introduce yourself by saying your name and your field of work and background?

---

3. Discussion (50 minutes - could be 5 minutes longer)

3.1. As you know, all our meetings will be dedicated to ethics. Today I'd like to talk about your understanding of ethics and opinion about it. I'd like to start from ethics in general.

I'll show you a word cloud made from your answers about what ethics means to you.

PPT first slide: ethics word cloud

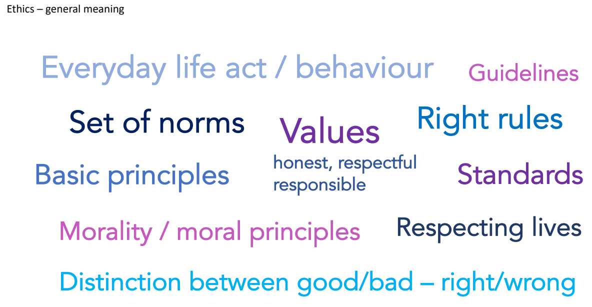

You can see your answers and what other said. Now I'd like to discuss **how you understand ethics and what meaning of this concept from the slide is closer to your understanding**.

3.1a. Would like to add anything to this slide?

3.2. What examples of ethics in daily life situations come to your mind?

3.3. Let's watch together this video: **https://www.youtube.com/watch?v=u399XmkjeXo**
What`s new for you after this video? Was there something we haven't discussed today?

---

5. Ending the discussion (1 minute)

Do you want to add something or ask any question?

Thank you so much for your engagement and your interesting thoughts in our discussion. I do appreciate it. Thank you for very useful contributions.

It will be very helpful for building a framework of good science and research. We will continue discussing about similar topics and as follow up next time.

Let me remind you that this data will be storage in a safe manner.

See you soon!

2st FOCUS GROUP GUIDE

---

1. Scene setting and ground rules (5 minutes)
1.a. Personal introduction:

My name is Paola, as you know, and I´m an Early-Stage Researcher in CARTHAGO project as all of you. Thank you so much for being here, and I'm looking forward to discussing and exchange ideas and thoughts with you. Ida will stay with us doing the technical support, so if you had any problems send her direct message and she will help you.

1.b. Outline of the research topic and purpose of the study:

Our meeting is part of one of the work packages of CARTHAGO: 2.5.

Work Packages 2.5 is about responsible research and innovation, following Horizon 2020 aims. The idea is to establish an educational framework towards the integration of ethics in research. Ethical issues of a novel biomedical intervention are identified and evaluated parallel to development of the field, rather than at the end-of-pipeline

The plan is to have several and periodical meetings to think, exchange ideas, debate different issues related to ethics.

1.c. Motivation to participate:

This meeting is for us, for all of us. As Work Package established, in these meetings we have to think about ethical issues on our research process, from our perspective. The important issue is that the perspective that matters is ours. And the framework that we start to build here will help us to be better scientists.
Also, what we start doing today is an innovative way of practice bioethics, so we are also being part of something kind of Avant Gard.

1.d. Ethics, confidentiality, and data process:

All these discussions were approved by the ethics committee of the Jagiellonian University Medical College, Cracow, Poland.

We will record this meeting. Recordings will be used only for research purposes. Data will be stored in safe place, following the EU General Data Protection Regulation (GDPR), as you already know after signing GDPR and inform consent form.

1.e. How to participate in the right way? Structure and rules

How our meetings will look like: This is an open discussion. I will ask questions, show you videos and pictures to start our conversation. You should feel free to say what you think, to present doubts that you may have, or to share some experiences and comments. We are on a safe environment here. It is important to know that there are no right or wrong answers. All points of view are important and welcomed. It is also important to agree or disagree with other participants because our goal is to hear as many as thoughts as possible.

Practical rules:
- Try to participate in every topic.
- Always talk to the group and not to each other in sub-groups.

- Raise virtual hand every time you want to say something - this will help us to organize the discussion better.

Do you have any doubts or questions?

---

2. Discussion (40 minutes – could be 10 minutes longer)

We will start with research ethics today, also in a general way.

2.1. What do you think, which research topics need to be ethically considered or on what elements of research process ethics should be applied? Why?

2.2. I will share with you the word cloud with your answers about ethical issues in scientific work. What meaning of the slide is closer to your understanding? Would you like to add anything to the slide?

PPT first slide: research ethics word cloud

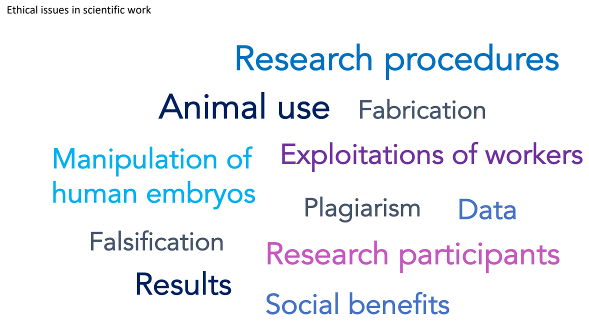

2.3. What do you know about Responsible Research and Innovation (RRI)?

2.4. Is there any relation between research ethics and RRI? Why?

2.5. Do you hear about the ALLEA (All European Academies) Code of Conduct for Research Integrity? The European Commission recognises the Code as the reference document for research integrity for all EU-funded research projects and as a model for organisations and researchers across Europe.
The European Code have stablished 4 fundamental principles of research integrity:

PPT second slide: 4 principles of RRI

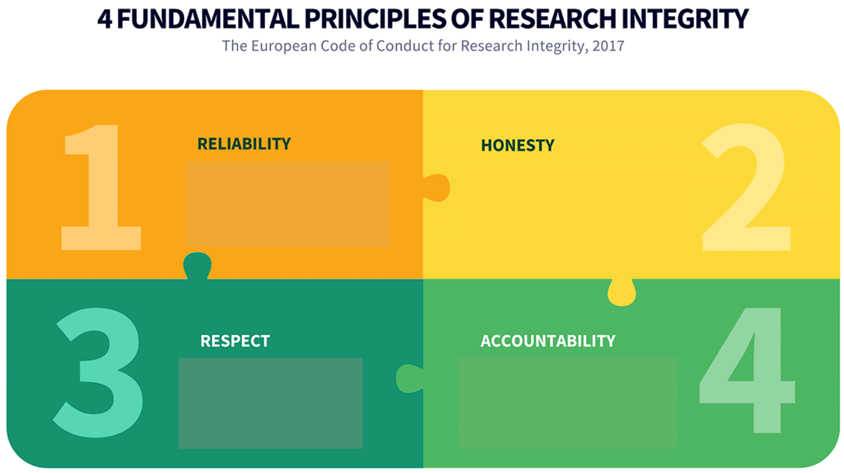

Can you describe and give an example of each of them? Let’s do it on Jamboards

Jamboards

Now, I will share with you the official definitions of that principles:

PPT third slide: definitions of 4 principles of RRI

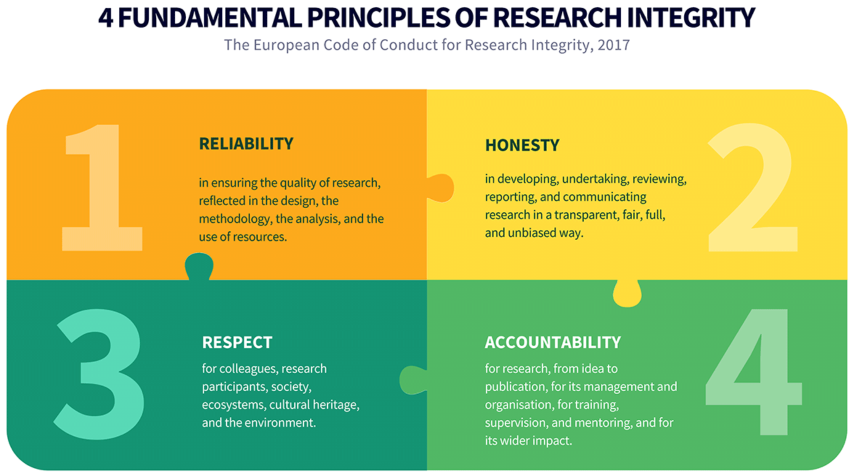

2.6. Considering these are the principles of good practices, what do you think are the ethical violations of these principles?

Wait for answers

Let’s take a look on these ethical violations:

PPT fifth slide: 3 ethical violations

- Fabrication: The creation of non-existant data and results and the act of recording and reporting them.

- Falsification: The manipulation of research materials, equipment or preocesses or omitting data and results so that the research is not accurately represented in the research record.

- Plagiarism: The appropriation of another person's ideas, processes, results or words without giving the appropriate credit.

2.7. What's you guess – are these violations frequent or not?

2.8. Why are these actions not supposed to be done? What are the consequences of this actions?

*In case we have time:

Considering all what we have discussed today: How can we think about the relation between ethics in daily life and ethics in research?

How do you perceive relation between legal regulations or institutional norms about research and research ethics?

---

3. Ending the discussion (1 minute)

Do you want to add something or ask any question?

Thank you so much for your engagement and your interesting thoughts in our discussion. I do appreciate it. Thank you for very useful contributions.

It will be very helpful for building a framework of good science and research. We will continue discussing about similar topics and as follow up next time.

Let me remind you that this data will be storage in a safe manner.

See you soon!

3rd FOCUS GROUP GUIDE

---

1. Scene setting and ground rules (2 minutes)

Thank you so much for being here, and I'm looking forward to discussing and exchange ideas and thoughts with you. Ida will stay with us doing the technical support, so if you had any problems send her direct message and she will help you.

Our meeting is part of one of the work packages of CARTHAGO: 2.5., following Horizon 2020 aims. This meeting is for us, for all of us. As Work Package established, in these meetings we should think about ethical issues on our research process, from our perspective. And this is the most valuable thing: that is done from within the biomedical research.

All these discussions were approved by the ethics committee of the Jagiellonian University Medical College, Cracow, Poland. We will record this meeting if you all agree with that. Recordings will be used only for research purposes. Data will be stored in safe place, following the GDPR.

Remember: you should feel free to say what you think, to present doubts that you may have, or to share some experiences and comments. We are on a safe environment here. All points of view are important and welcomed. It is also important to agree or disagree with other participants because our goal is to hear as many as thoughts as possible.

Practical rules:
- Try to participate in every topic.
- Always talk to the group and not to each other in sub-groups.
- Raise virtual hand every time you want to say something - this will help us to organize the discussion better.

Do you have any doubts, comments, or questions?

---

2. Discussion

On our first meeting we talked about ethics, in a general way. On our second meeting we approached research ethics, also in a general way.

The main research topic of CARTHAGO has to do with human gene transfer and regenerative medicine for disc and joint pathology. Today, we are going to debate about the ethical aspects of the topic, to be able to know about them and work on them. This is our responsibility as WP2.5. We need to make sure that all what is being develop is ethically acceptable.

Part 1: Individual perspective (30 minutes)

So, let's visualize this:

Slide 1

Maybe you remember that you completed a questionnaire before we started this focus groups meetings. And maybe you remember that there were two questions about potential ethical challenges about non-viral gene therapy and orthopaedic regenerative medicine that. I will show you your answers:

Slide 2 and 3

2.1. It is quite balance, right? So let's talk about it. Why do you think there could be potential ethical challenges about non-viral gene therapy and why not?

2.2. Let's summarize – what ethical issues you could indicate in this case?
Slide 4 that I will complete while they talk Would you like to add something to what I wrote on the slide?

2.3. As researchers, what is your greatest worry in the development process of this topic?

2.4. What do you think, how personal or individual bias can affect the development process of this topic?

Part 2: Global perspective (40 minutes – could be 10 minutes longer)

2.5. Which do you think is the main societal value of this topic?

2.6. How could society be impacted by this topic?

2.5. So now I'd like to ask you to work in small groups. We are going to look CARTHAGO main goal through specifics values. Do you remember the Responsible Research and Innovation (RRI) framework? Horizon 2020 and our Work Package aims to work under. Indeed, it is the name of our WP.

Slide 5

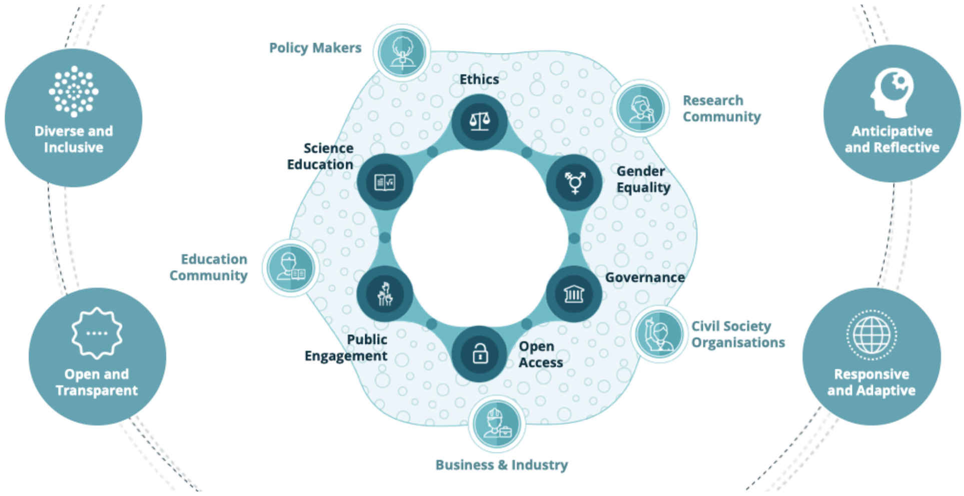

You can see here that there is an inner circle, which is the heart of the RRI, then society actors that should be involved in RRI, and surrounding all that, there are 4 big circles of values.

Slide 6

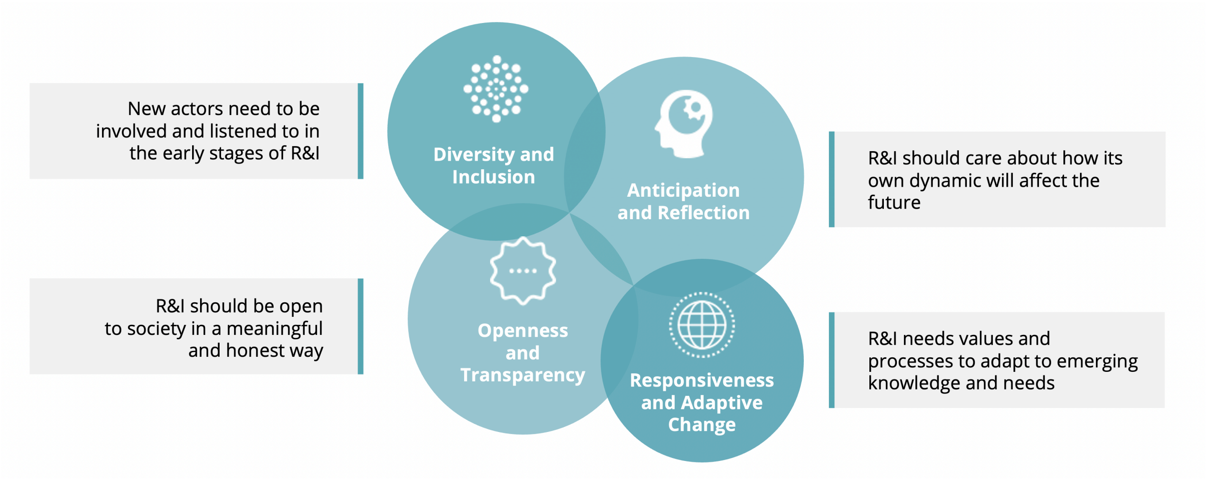

Each group will work with one big circle of values. I'd like you to think and discuss in your group – how CARTHAGO topic (human gene transfer and regenerative medicine for disc and joint pathology) could be related to that value, in a good or bad way and then to write your ideas in the Jamboard.

You have 10-15 minutes to discuss and write your suggestions. If you have any questions you can write to me on chat.

Split groups and Jamboards

Ok, so we are back. Let's share and discuss what you have done. One per group explain and then we can make questions, comments or add other thoughts to the Jamboard.

---

3. Ending the discussion (1 minute)

Well, it has been an enriching meeting.

Do you want to add something or ask any question?

I will send you material about RRI, todays Jamboards and the one that you did in the last FG. Did you receive what I sent before?

Thank you so much for your engagement and your interesting thoughts in our discussion. I do appreciate it. Thank you for very useful contributions. See you in April!

4th FOCUS GROUP GUIDE

---

1. Scene setting and ground rules (2 minutes)

Thank you so much for being here, and I'm looking forward to discussing and exchange ideas and thoughts with you. Ida will stay with us doing the technical support, so if you had any problems send her direct message and she will help you.

Our meeting is part of one of the work packages of CARTHAGO: 2.5., following Horizon 2020 aims. This meeting is for us, for all of us. As Work Package established, in these meetings we should think about ethical issues on our research process, from our perspective. And this is the most valuable thing: that is done from within the biomedical research.

All these discussions were approved by the ethics committee of the Jagiellonian University Medical College, Cracow, Poland. We will record this meeting if you all agree with that. Recordings will be used only for research purposes. Data will be stored in safe place, following the GDPR.

Remember: you should feel free to say what you think, to present doubts that you may have, or to share some experiences and comments. We are on a safe environment here. All points of view are important and welcomed. It is also important to agree or disagree with other participants because our goal is to hear as many as thoughts as possible.

Practical rules:
- Try to participate in every topic.
- Always talk to the group and not to each other in sub-groups.
- Raise virtual hand every time you want to say something - this will help us to organize the discussion better.

Do you have any doubts, comments, or questions?

---

2. Discussion (80 minutes)

Introduction (15 minutes)

In the first meeting we focused on ethics in a general. In the second - we narrowed down our subject and approached research ethics. When met third time we debated about the ethical aspects of human gene transfer and regenerative medicine for disc and joint pathology.

Today, we are going to deepen our subject again and work on the ethics of methods used in our work packages (WP):

Slide 1
2.1. Cell delivery and efficiency gene modulation
2.2. Tissue/organ delivery tools
2.3. Repair in tissue and organ culture
2.4. In vivo imaging of regeneration and gene therapy efficacy

In the questionnaire, some of you shared that your methods could have potential ethical issues. So, for the start I'd like to ask you if or when you had a chance to think about the ethical aspects of the methods or techniques that you are using?

[GROUP DISCUSSION – 10 minutes]

Today we are going to work in small groups. Each group will have to ethically analysed each WP and it's methods and show them in a visual way using MIRO boards.

Do you have any question so far?

1st task (15 + 15 minutes = 30 minutes)

So let's start with the first board. The goal is to communicate how the WP and it's methods -the ones that you think it's important to consider- could have positive impact in some areas. I prepare for you examples of areas that may be influenced by methods of your WP:

Slide 2 and 3

- respect for human agency: autonomy and dignity
- privacy and personal information
- social well-being
- health inequalities
- mental health
- biodiversity
- climate change
- ageing population
- increased urbanization

You have 15 minutes to prepare the board in your group. Then we'll meet to briefly present what you have done – each group will have about 3 to 5 minutes for presentation and the comments from the rest of the group.

Now I'd like to ask you to click on the link on chat and go to MIRO board. When you are there, please click on the small arrow next to the name of your group. It will take you to your group board.

I will assign you to separate rooms where you can discuss. If you have any problems write on chat to Ida. See you in 15 minutes.

[TASK I – 15 minutes]

So let's start from group one…

[DISCUSSION – 15 minutes]

2nd task (20 minutes + 15 minutes = 35 minutes)

Ok, thank you for your presentations, let's go to our second task. Now I would like to ask you two things - first, think about the negative impact the methods of the methods of your WP might have. You can use the dimensions from previous task. When we get to MIRO, you will find a place to describe the negative influence.

When you finish this, I'd like to ask you to prepare second board which addresses how you can prevent this negative influence and present the possible solutions of previously mentioned problems.

For those you have 20 minutes. Then we'll meet to present what you have done. Each group will have about 3 to 5 minutes for presentation and the comments from the rest of the group.

[TASK II – 20 minutes]

So let’s start from group one…

[DISCUSSION – 15 minutes]

---

3. Ending the discussion (3 minute)

Well, it has been an enriching meeting.

Do you want to add something or ask any question?

I will send you material about what we worked on today and the posters. Did you receive what I sent before?

Thank you for very useful contributions. See you in May!

5th FOCUS GROUP GUIDE

INTRODUCTION

1. Scene setting and ground rules (2 minutes)

Ida will stay with us doing the technical support, so if you had any problems send her direct message and she will help you.

Practical rules:
- Try to participate in every topic.
- Always talk to the group and not to each other in sub-groups.
- Raise virtual hand every time you want to say something - this will help us to organize the discussion better.

Do you have any doubts, comments, or questions?

---

2. DISCUSSION (75 minutes)

Part 1

As this is our last focus group meeting, I prepared for you a short sum-up of what we did:

Prezi

Are there aspects which currently you see as the more important than others? Which ones? Why? Explain, please give examples.

Is there anything that you would like specially to highlight or comment?

If you have to tell someone else what was the most meaningful part of our discussions, what would you say?

+ mention that they will have a chance to write more in questionnaire

How you see the importance and role of research ethics?

Part 2

Today, we are going to formulate recommendations for integrating ethics to research in international biomedical projects.

The recommendations should lay on how to improve research from an ethical point of view and considering all the discussions that we had in all our meetings. Of course, you can also come up with something new.

The recommendations should be formulated following two perspectives:
1. What you can do: focus on what ESRs could do/change.
2. What should be done on an institutional level: focus on what the research group, PI, university, or states could do/change.

We are going to work in small groups according to the WP like last meeting.

You have 20 minutes to prepare recommendations. I prepare for you Jamboards with the name of your WP and the two perspectives of recommendation. I will assign you to separate rooms where you can discuss.

[TASK – 20 minutes]

So, let's start from group one…

[DISCUSSION – 30 minutes]

*Please comment which recommendations you find as **must** which ones are **optional**. Why these?*

With which recommendations you agree and with which you could hesitate?

How do you feel as competent in applying this ethics recommendations to the research? Are there practical? Feasible? Possible? What kind of challenges still exist? What kind? How to solve them?

---

3. CLOSE (3 minutes)

Thank you, it has been a productive meeting.

Do you want to add something or ask any question?

Ok, so this was our last meeting, I hope you enjoyed all of them, and you learn something from these meetings.

To finish this process, I will send you a post-focus group questionnaire, and there you can assess and make an opinion or critics on what we did here.

Thank you for very useful contributions. See you in Davos!
